# Simulating respiratory disease transmission within and between classrooms to assess pandemic management strategies at schools

**DOI:** 10.1073/pnas.2203019119

**Published:** 2022-09-08

**Authors:** Akira Endo (遠藤彰), CMMID COVID-19 Working Group, Mitsuo Uchida (内田満夫), Yang Liu (刘扬), Katherine E. Atkins, Adam J. Kucharski, Sebastian Funk

**Affiliations:** ^a^Department of Infectious Disease Epidemiology, London School of Hygiene & Tropical Medicine, London WC1E 7HT, United Kingdom;; ^b^The Centre for Mathematical Modelling of Infectious Diseases, London School of Hygiene & Tropical Medicine, London WC1E 7HT, United Kingdom;; ^c^The Alan Turing Institute, London NW1 2DB, United Kingdom;; ^d^School of Tropical Medicine and Global Health, Nagasaki University, Nagasaki 852-8523, Japan;; ^e^Japan Society for the Promotion of Science, Tokyo 102-0083, Japan;; ^f^Graduate School of Medicine, Gunma University, Gunma 371-8511, Japan;; ^g^Centre for Global Health, Usher Institute, University of Edinburgh, Edinburgh EH16 4UX, United Kingdom

**Keywords:** influenza, school, mathematical model, class size, social network

## Abstract

The global spread of coronavirus disease 2019 (COVID-19) has emphasized the need for evidence-based strategies for the safe operation of schools during pandemics that balance infection risk with the society’s responsibility of allowing children to attend school. Due to limited empirical data, existing analyses assessing school-based interventions in pandemic situations often impose strong assumptions, for example, on the relationship between class size and transmission risk, which could bias the estimated effect of interventions, such as split classes and staggered attendance. To fill this gap in school outbreak studies, we parameterized an individual-based model that accounts for heterogeneous contact rates within and between classes and grades to a multischool outbreak data of influenza. We then simulated school outbreaks of respiratory infectious diseases of ongoing threat (i.e., COVID-19) and potential threat (i.e., pandemic influenza) under a variety of interventions (changing class structures, symptom screening, regular testing, cohorting, and responsive class closures). Our results suggest that interventions changing class structures (e.g., reduced class sizes) may not be effective in reducing the risk of major school outbreaks upon introduction of a case and that other precautionary measures (e.g., screening and isolation) need to be employed. Class-level closures in response to detection of a case were also suggested to be effective in reducing the size of an outbreak.

## Significance

Interventions to control coronavirus disease 2019 (COVID-19) in school settings often assume that simply limiting the number of students attending reduces the potential for disease spread. However, using a mathematical model parameterized with a detailed dataset of seasonal influenza in Japanese primary schools, we find that interventions that focus only on reducing the number of students in class at any moment in time (e.g., reduced class sizes and staggered attendance) may not be effective. We propose two approaches for pandemic management in school settings: a routine “preemptive” approach that attempts to keep the within-school reproduction number low by, for example, regular screening and cohorting and a “responsive” approach where fixed-period class closures are employed upon detection of a symptomatic case.

With the emergence and rapid growth of the coronavirus disease 2019 (COVID-19) outbreak in early 2020, many countries introduced school closures to prevent schools from becoming hot spots of transmission and mitigate the further spread in the population (1, 2). Worldwide, balancing ongoing control measures and access to education has been challenging and often controversial. Many regions reopened schools by late 2020, employing a range of precautionary measures, such as universal masking, increased ventilation, enhanced hygiene, reduced class sizes, and “cohorting” (i.e., limiting contacts to small groups of students; also referred to as “social bubbles”) (3, 4). Nonetheless, school policies for outbreak control have substantially varied both geographically and over the course of the pandemic (2). Such diverse and dynamic political decisions in part reflect limited and time-evolving understanding of the relative role of schoolchildren in the transmission of severe acute respiratory syndrome virus 2 (SARS-CoV-2). While most infections are mild or asymptomatic among children (5–8) and their susceptibility to infection has been suggested to be lower than that of adults (9–12), a growing body of evidence has shown that children can still contribute to transmission (1, 13–18). Infectiousness of children may be similar to that of adults (19, 20), although uncertainties in currently available evidence remain (10, 11). Reports of COVID-19 outbreaks in school settings had been relatively rare in the earliest phase of the pandemic, including while schools were fully open (21–26); however, these data need to be interpreted with caution as multiple factors, including asymptomatic infections, variability in transmission, and enforcement of precautionary measures, could have been involved. There have been sporadic reports of large outbreaks associated with schools in various countries (27–29). Moreover, with the progress of vaccination for adults and the emergence of more transmissible variants, the focus of transmission has gradually shifted to the younger population, and their relative roles were suggested to have become greater (1, 30–33).

Decisions about whether and how to keep schools open during a pandemic need to weigh the rights and welfare of children and their families against the public health implications. Although epidemiological aspects of such policies should ideally be informed by scenario analyses using mathematical models, supporting data on detailed within-school transmission dynamics and possible effects of interventions in school settings are relatively scarce. As a result, many existing modeling studies that assess the effect of school opening strategies for COVID-19 have three important limitations (34–40). First, they mostly assume that the contact rates of students are proportional to the number of students attending (at class or at school), which is not empirically validated. This assumption of density-dependent mixing entails that reducing student attendance (e.g., by introducing small class sizes or staggered attendance) would linearly scale down the transmission risk, which may overestimate the effect of interventions. Second, while school-based interventions aim to reduce social contact within and between classes and grades, previous studies have not been able to parameterize the corresponding contact rates from empirical data and impose strong assumptions on the relative contributions of these contacts. Third, previous studies on parameters related to within-school transmission dynamics were based on a limited number of schools and thus, did not account for differential mixing patterns across schools or capture the full range of heterogeneity present (41, 42).

To overcome these limitations and provide a more robust assessment of school-based interventions, we parameterized a within-school transmission model that considers social contacts within and between classes and grades to a detailed dataset of seasonal influenza from 29 primary schools with a total of over 10,000 students (43). This calibration allowed us to capture granular social contact patterns relevant to empirical transmission dynamics of respiratory virus in schools, including the correlations between transmission patterns and class/school sizes, although with a caveat of the dataset being observational. We then rescaled the estimates to the assumed within-school reproduction numbers and combined them with the temporal infection profile of SARS-CoV-2. Using this simulation model, we assessed the risk and size of outbreaks under current COVID-19 interventions in wide use, such as changes in class structure, screening and isolation, cohorting, and responsive class closures. We conducted sensitivity analysis by also adapting the model with possible transmission parameters of pandemic influenza in order to assess the robustness of these optimal strategies to a different pathogen.

## Results

### Infection Profile of SARS-CoV-2 and the Effect of Screening.

**Fig. 1.**
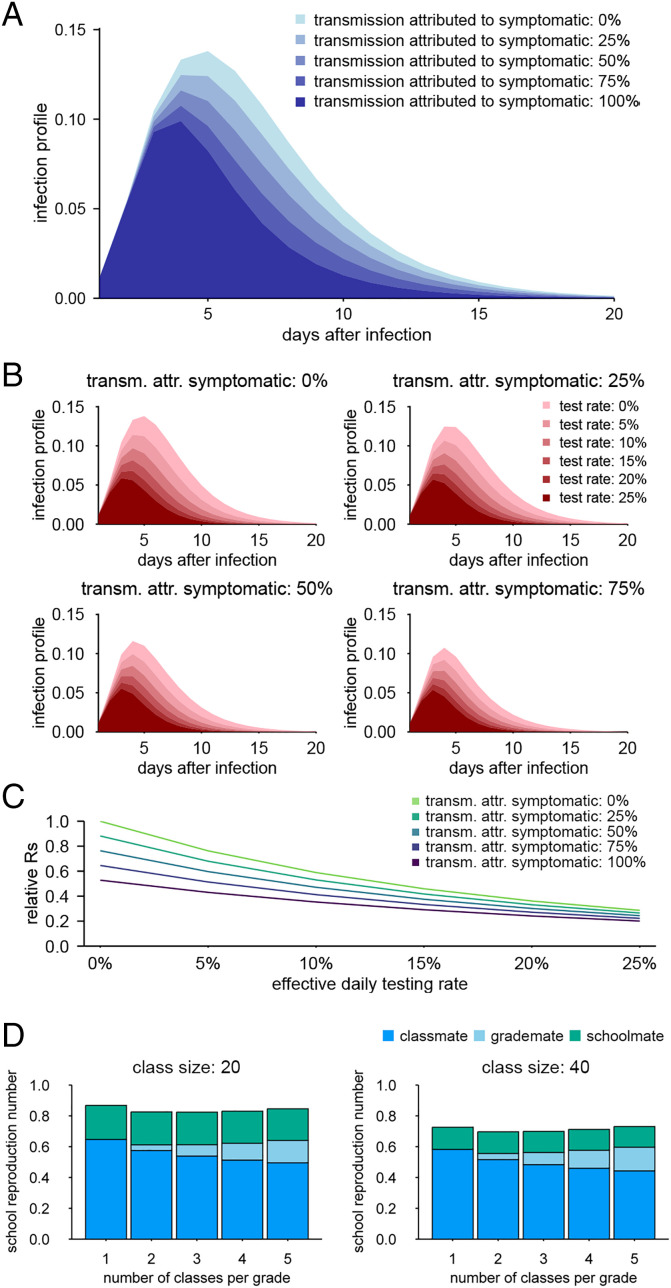
Time-dependent infection profile of SARS-CoV-2 and the possible effect of screening. (*A*) The effective infection profile for various symptomatic proportions, where symptomatic students are isolated from the next day after the symptom onset and do not contribute to further transmission (symptom screening). (*B*) The effective infection profile where students are screened by both symptoms and regular tests. Colors represent daily effective testing rates. Students are assumed to be isolated from the next day after presenting either symptoms or from the day of a positive test result. (*C*) The relative change in the reproduction number with combinations of symptoms and regular test screening. (*D*) Estimated school reproduction number (*R*_S_) for seasonal influenza for different class sizes and the number of classes per grade. Breakdowns by the source of infection are shown: from within the class (classmates), from outside the class but within the same grade (grade mates), and from outside the grade (schoolmates). Adapted from ref. 43, which is licensed under CC BY 4.0.

We reconstructed the time-dependent infection profile (i.e., time course of the transmission potential as a function of time after infection) of SARS-CoV-2 from distributions estimated in the literature (44) and assessed the possible reduction in the within-school reproduction number (*R*_S_) by screening either by symptoms or by regular testing (Fig. 1). If every student showing COVID-19–like symptoms is asked to isolate, postsymptomatic transmission within the school will be prevented. Postsymptomatic transmission is estimated to account for around 60% of the total secondary transmission of symptomatic individuals (44) and therefore, expected to suppress the right tail of the infection profile. However, since symptom-based isolation will not apply to asymptomatic infections, the proportion of preventable transmission also depends on the relative contribution of symptomatic infections to onward transmission (referred to as transmission attributable to symptomatic infections in Fig. 1). This is shaped by the frequency and infectiousness of symptomatic vs. asymptomatic infections; for example, if 50% of infections are asymptomatic and their relative infectiousness is 50% of that of symptomatic infections, 67% of onward transmission would be attributed to symptomatic infection. The evidence on these epidemiological properties remains inconclusive (8, 45–47), and that for children is scarce. One multicenter cohort study estimated that 50% of seropositive children aged 2 to 15 y had been symptomatic (48). The performance of symptom screening could be even lower if some mild/atypical symptoms were missed in screening; only 38% of seropositive children in the above study exhibited typical COVID-19 symptoms (fever, cough, or changes in smell/taste). Combined with the estimated relative reduction from none to up to 60% in infectiousness of asymptomatic infections (45), transmission from ever-symptomatic students may account for 38 to 71% of secondary transmission.* Symptom screening alone may reduce the reproduction number by about 20 to 40% in such a setting (Fig. 1*C*). In the later simulation, we chose 50% as the baseline assumption for the proportion attributed to symptomatic students.

In addition to symptom screening, we also considered screening by regular (rapid) testing, which we assumed to detect infectious individuals at a given probability (sensitivity) from the second day of infectiousness. The daily rate of infectious students detected by a test (who will be asked to isolate from the day of positivity) is given by the product of the frequency and sensitivity of the test (“effective daily testing rate”). Combined with symptom screening, regular testing could further reduce the risk of transmission (Fig. 1*B*). Of note, we estimated a 10 to 20% daily effective testing rate (roughly corresponding to performing a 70% sensitive test once or twice a week for every student) to be sufficient to reduce the reproduction number by 40 to 70%, and the effect saturates after the daily effective testing rate exceeds 15% (Fig. 1*C*). Such testing frequency is similar to that of the regular testing scheme for students of UK schools that started in 2021 (49), where students were recommended to undertake twice-weekly home testing using lateral flow kits.

### Simulation of COVID-19 Outbreaks in Schools.

**Fig. 2.**
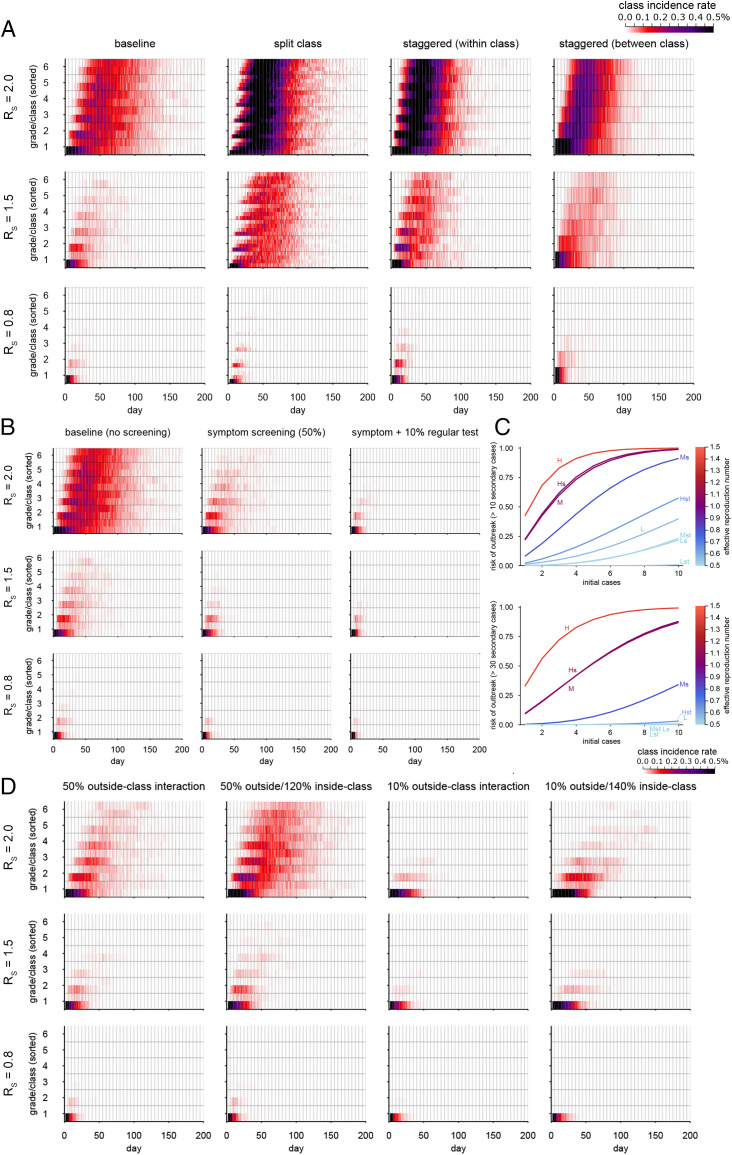
Outbreak simulations of SARS-CoV-2 in six–year group primary schools under interventions. (*A*) Simulated temporal patterns of outbreaks under interventions changing class structures. Colors represent the mean class incidence rate (the number of new infections on a single day in each class divided by the class size) over the 500 simulations. For each simulation, grades and classes are sorted by the date of the first case in the class so that the spread of infections in classes is time ordered from the bottom to the top. (*B*) Simulations with symptom screening and regular testing. (*C*) The estimated risk of large outbreaks with multiple introductions. Curves show the probability that the eventual number of secondary transmissions within the school exceeds 10 or 30 cases in the intervention scenarios, given multiple introductions of infected students from outside the school. Interventions are labeled by the following notations. H indicates that the school reproduction number (*R_S_*) is 2.0, M indicates that *R_S_* = 1.5, L indicates that *R_S_* = 0.8, s indicates that screening is by symptoms, and t indicates that screening is by regular testing (effective rate 10%). Colors denote the effective reproduction number within the school for each intervention. (*D*) Simulations with reduced outside-class interactions (class cohorting). Compensatory increases in the within-class interactions (20 and 40% increases in within-class interactions to compensate for 50 and 90% reductions in outside-class interactions, respectively) were also considered as part of the simulation.

**Fig. 3.**
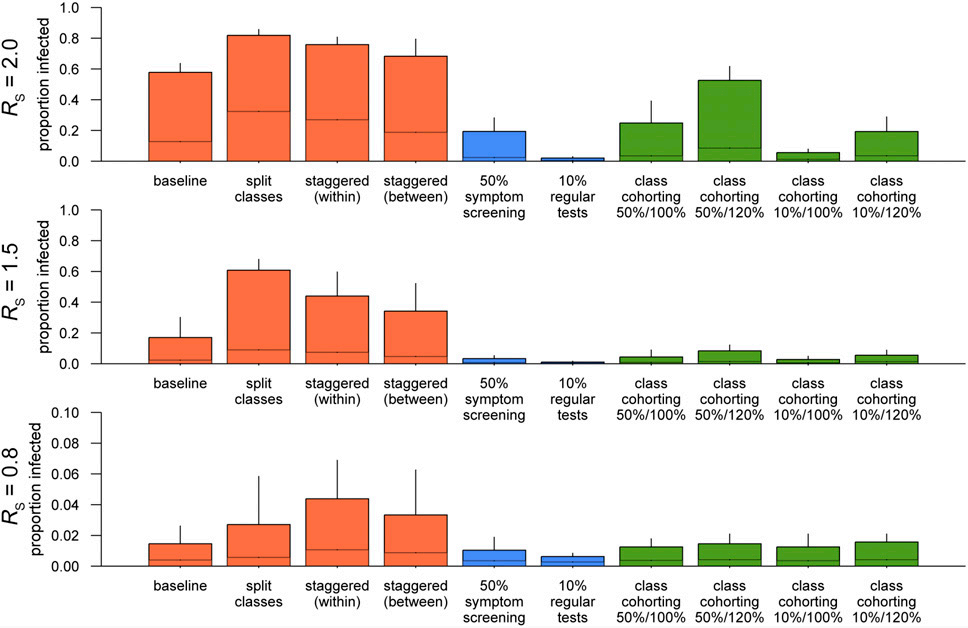
The distribution of simulated final sizes of COVID-19 school outbreaks under interventions. Bars represent the upper 95% bounds, and the middle lines show the means over the simulations. Whiskers denote the upper 99% bounds. Colors represent different categories of interventions: changing class structures (orange), screening and testing (blue), and class cohorting (green). Note that symptom screening is also assumed to be conducted in the “10% regular tests” scenario.

To examine the outbreak dynamics, we simulated the heterogeneous transmission of COVID-19 within and between classes and grades in a school with six year groups. We parameterized our simulation model using the estimates of within-school transmission patterns (i.e., the relative transmission risks between students within class, grade, and school) (Fig. 1*D*) of seasonal influenza in primary schools in Matsumoto City, Japan (43), assuming that both COVID-19 and influenza share a similar mode of transmission and spread over social contacts at school (50). By combining these transmission patterns with the initial infection profile of SARS-CoV-2 (known to have a longer course of infection than influenza), we simulated possible outbreaks of COVID-19 triggered by a single case introduced from outside the school (Figs. 2 and 3). We assumed three values (2.0, 1.5, 0.8) for the within-school reproduction number *R*_S_ as representative of the Delta-like, Alpha-like, and the original SARS-CoV-2–/flu-like transmission potential, respectively, based on the estimate of *R*_S_ = 0.8 for seasonal influenza (43) (*Materials and Methods* has more details). For each of these values, temporal patterns of disease spread across the school (Fig. 2) and the distributions of final outbreak size (Fig. 3) were compared between a variety of interventions (Table 1). We estimated, without interventions, that introduction of a case in a single class would be quickly followed by secondary cases in multiple classes and grades and that cases may be observed in most of the classes and grades 20 to 30 d after the introduction at the earliest in scenarios where *R*_S_ is above one. We also found that the simulated Delta-like outbreaks under the symptom screening intervention were of the same order of magnitude in size as the observed school outbreaks in Texas in the United States during the Delta variant predominance (*SI Appendix*, Fig. S8).

**Table 1.** List of intervention types explored in the simulation

Interventions	Description	Effect in the model
Changing class structures	Includes three different interventions that change the parameters *n* (class size) and *m* (no. of classes per grade) in the model as specified in Table 2; the baseline settings are *n* = 40 and *m* = 2	Changes parameters *n* and *m*
Symptom screening	Infected students were assumed to self-isolate from the next day after symptom onset	Modifies infection profile
Regular testing	Students receive regular tests, and those who tested positive were assumed to self-isolate from the day of receiving the positive test; regular testing is always combined with symptom screening in our simulation	Modifies infection profile
Class cohorting	The interactions between students from different classes were reduced; a possible increase in within-class interactions resulting from limiting outside-class interactions was also considered as part of the scenarios	Changes the force of infection for between-class transmissions
Responsive class closure	From the next day after symptom onset or the day of a positive test (when regular testing is in operation), all classmates of an infected student are held in quarantine for 10 d (COVID-19)/5 d (influenza)	No transmission to and from students in closed classes

Reflecting that the *R*_S_ for seasonal influenza was estimated to be mostly invariant between classes and schools of different sizes (43), interventions that changed the size or the number of classes (split class and staggered attendance) (Table 2) were not predicted to contribute to outbreak control (Fig. 2*A*); they may even increase the final outbreak size (Fig. 3). Screening by symptoms and regular testing was suggested to be effective in reducing outbreak sizes (Figs. 2*B* and 3); if infected students who become symptomatic at any point during their infectious period contribute to 50% of onward transmission, symptom screening alone could render the scale of an outbreak with *R*_S_ = 1.5 comparable with one with *R*_S_ = 0.8 with no intervention. A combination of symptom screening and regular testing (effective daily test rate of 10%) could even bring an outbreak with *R*_S_ = 2.0 to a similar level. Additional analysis using peak weekly incidence as the outcome measure suggested that the interventions that can reduce outbreak sizes were also effective in reducing peak incidence (*SI Appendix*, Fig. S2).

**Table 2.** Summary of interventions that change the size/number of classes (from ref. 43)

Interventions	Class size (*n*)	No. of classes per grade (*m*)	Assumption
Baseline (“no change”)	40	2	Students’ contacts within and between classes and grades are proportional to the estimated transmission patterns in Fig. 2
Split class	20	4	Each class is split into two and taught simultaneously in separate classrooms; students may contact each other between classes
Staggered attendance (within class)	20	2	Each class is split into two and taught separately in two different time slots (e.g., morning and evening); students in different time slots do not contact each other, and thus, *R*_S_ is calculated for students in one slot
Staggered attendance (between class)	40	1	Each class is allocated (as a whole) to either of the two different time slots and taught separately; students in different time slots do not contact each other, and thus, *R*_S_ is calculated for students in one slot

To simulate outbreaks with “class cohorting,” where between-class interactions are reduced (e.g., by enforcing social bubbles within classes), we employed two different assumptions on the change in within-class interactions. On one hand, within-class interaction may remain constant when interactions with students outside the class are restricted; on the other hand, it could also cause an increase in the within-class interaction to compensate for the reduction outside the class. The latter assumption of compensatory increase may be in line with our previous study (43), which suggested that the total number of within-grade transmission per primary case was nearly constant regardless of the number of classes. While class cohorting was suggested to be generally effective in reducing outbreak sizes, its performance appears to be limited in settings with a low reproduction number or a compensatory increase (Figs. 2*D* and 3). For each intervention scenario, we also estimated the risk of outbreaks involving more than 10 or 30 secondary transmissions given multiple introductions of cases from outside the school (Fig. 2*C*). The results implied that when multiple introductions are expected due to high levels of community transmission, the risk of observing a large outbreak is substantially elevated. Our results suggest that the within-school reproduction number *R*_S_ needs to be kept around 0.5 or lower by routine school interventions in order to keep the risk of a large outbreak involving over 10 secondary cases at school at most around 20%; otherwise, additional responsive measures, such as class closure, may be required to prevent the spread. With this level of *R*_S_, the risk of an outbreak involving over 30 secondary transmissions becomes almost negligible. The risk of large outbreaks decreases if we assume a high degree of overdispersion in the individual-level transmission as is observed with general outbreaks of SARS-CoV-2 (51) (i.e., an overdispersion parameter of 0.2); however, assuming the same threshold for an acceptable risk (i.e., ∼20% risk of up to 10 secondary transmissions given 10 introductions), *R*_S_ of 0.5 or below should remain the primary target of routine school interventions (*SI Appendix*, Fig. S4).

### Managing School Outbreaks of COVID-19 by Single-Class Closures.

**Fig. 4.**
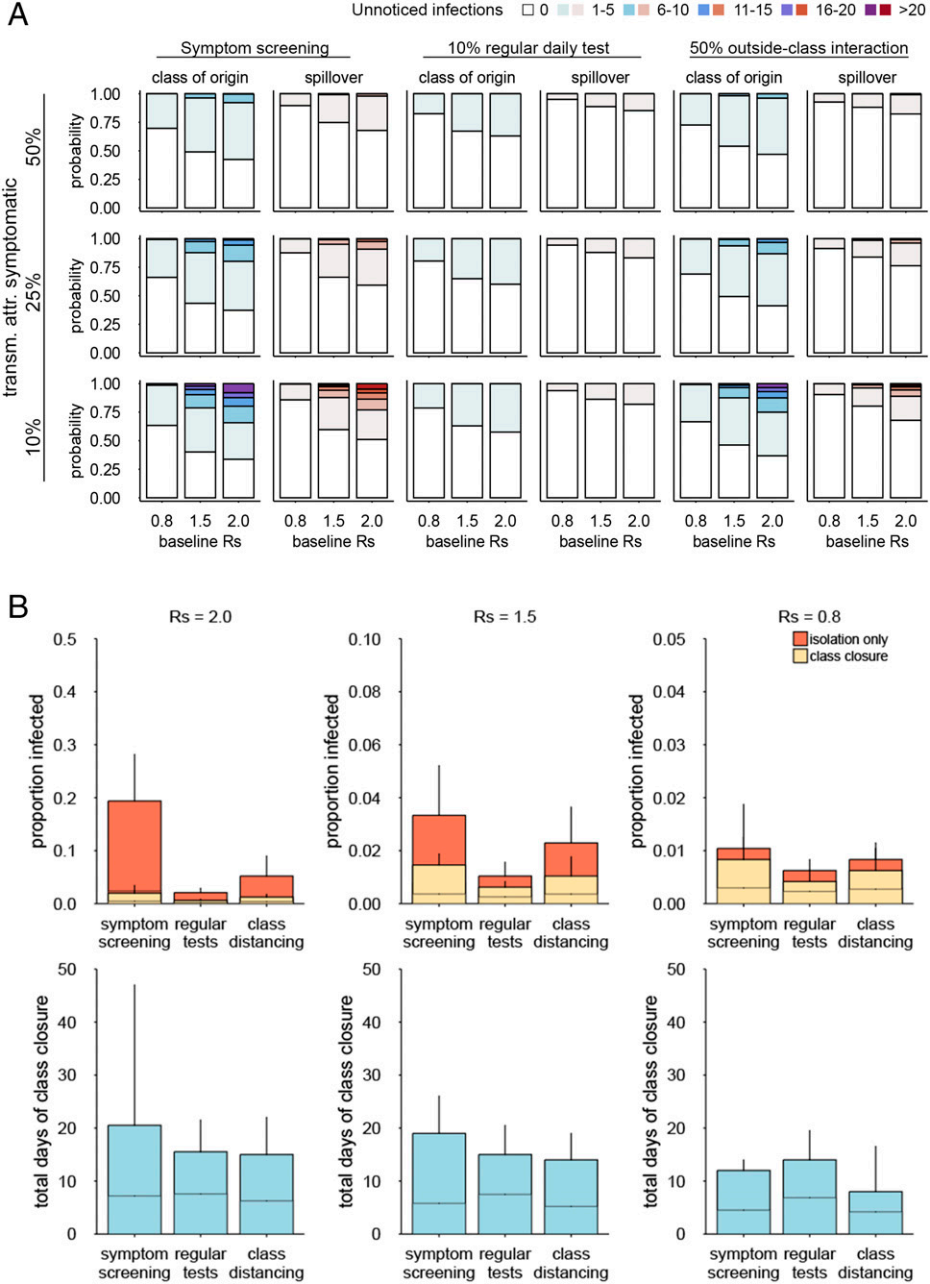
Likely scales of COVID-19 outbreak at the recognition of a case and simulations of single-class closure strategies. (*A*) The predicted distributions of the number of undetected infections by the time of the first identification of a case in school: overall (blue) and outside the class of the initial case (red; spillover). (*B*) The final size of simulated outbreaks with and without single-class closure strategies and the total days of class closures. (*Upper*) Comparison of the cumulative number of infections with and without class closures in each setting. (*Lower*) The distribution of the number of days of class closures aggregated across the school. Bars represent the upper 95% bounds, and the middle lines show the means over the simulations. Whiskers denote the upper 99% bounds. Note that *y* axes have different scales in *Upper*.

In addition to the above “preemptive” approaches (i.e., interventions that are initiated before the introduction of any cases), we also considered the use of “responsive” class closures, where students are asked to quarantine upon detection of a case in the same class. Whether such a responsive approach can be effective depends on how many transmissions would occur before the first case is recognized. We explored the conditions that allow for effective control by class closures by quantifying the number of undetected infections by the time a case is first recognized either by symptoms or by regular tests (Fig. 4*A*). If the case finding depends only on symptoms, it is likely (∼50% or more) that there has been at least one undetected infection before the first case is recognized. Moreover, in the case of *R*_S_ > 1, there is a chance of around 25 to 50% that undetected infections also exist outside the class of the first detected case (“spillover”), which suggests that closure of that class alone may be insufficient for containment and that subsequent infections can continue in other classes. If the proportion of transmission attributable to symptomatic infections is lower than our baseline assumption of 50%, the outbreak could reach a substantial size (even over 10 or 20 infections) by the time the first case shows symptoms. Introducing regular testing at the effective daily testing rate of 10% (corresponding to weekly testing with 70% sensitive tests) could markedly reduce the chance of undetected spread. The risk of outside-class spillover by the time of detection is limited to around 10%, which opens a possibility for control by closing only one class (or a few additional classes in the case of a rare spillover). If regular testing is not available and thus, case finding needs to depend on the presence of symptoms, another possible option is to implement class cohorting well before an outbreak is recognized to reduce the risk of spillover upon detection. When 50% of transmissions are attributable to symptomatics, reducing outside-class interaction by 50% is predicted to render the spillover risk comparable with the 10% daily testing scenario.

To quantify the resulting outbreak size and the associated loss of opportunity for education, we simulated outbreaks of COVID-19 in schools where the single-class closure strategy is in operation (i.e., a class is closed for 10 d if any student in the class is found to be infected [either by symptoms or a positive test], while other classes with no detected infection keep operating) (Fig. 4*B* and *SI Appendix*, Fig. S3*A*). Although the single-class closure strategies were suggested to be effective in reducing the outbreak size across the settings considered, the “symptom screening” strategy (i.e., a class is closed only if any of its students are found to be symptomatic) with no regular testing or class cohorting tended to result in a slightly larger outbreak and more class closures. Incorporating regular testing or class cohorting showed generally better performance with respect to both outbreak containment and education opportunities. However, combining regular testing with single-class closures warrants caution because the expected outbreak size is already small with 10% regular tests, and the additional benefit with class closure may be marginal given the associated 10- to 20-d loss of education opportunities (Fig. 4*B*). These results suggest that when regular tests are available, asking only test-positive students to isolate may be preferable to a class closure. Regular testing can identify infected students early in their infectiousness period; therefore, it becomes more likely that isolation alone is sufficient to prevent further transmissions.

### Simulation of Pandemic Influenza Outbreaks in Schools.

As a sensitivity analysis, we applied our pandemic management approaches discussed as above in the context of COVID-19 to another potential global health threat—pandemic influenza. Compared with COVID-19, influenza tends to exhibit a shorter time course (i.e., shorter generation time and incubation period), which may affect the effectiveness of screening by symptoms/regular tests. Although empirical data are relatively scarce on the symptomatic ratio of past pandemic influenza strains, that of seasonal H1N1 type A or H3N2 type A influenza strains in primary school-age children has been estimated to be around the range of 25 to 50%.

**Fig. 5.**
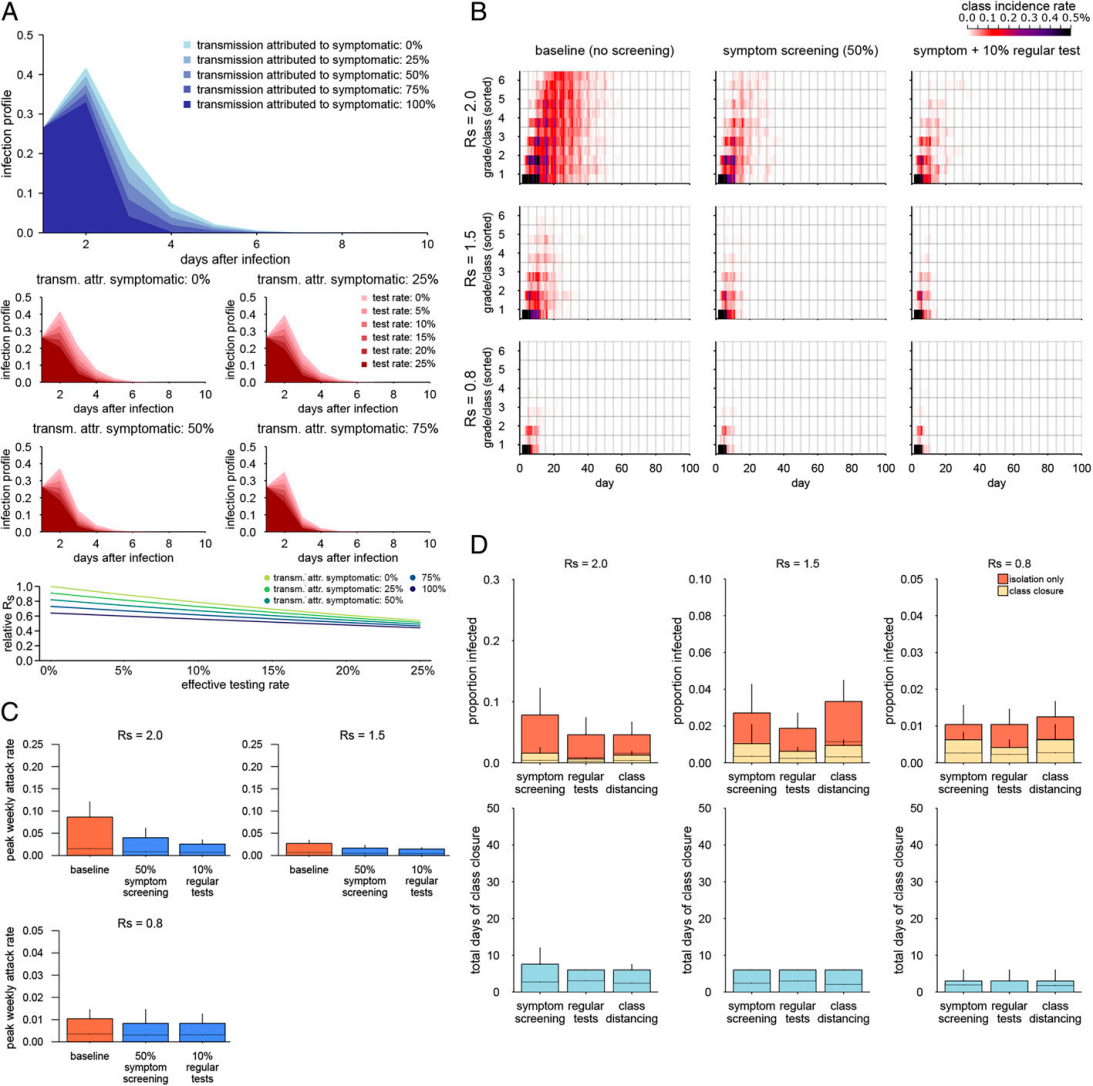
Simulated patterns of pandemic influenza outbreaks in schools. (*A*) The assumed time-dependent infection profile of pandemic influenza and possible reduction by screening. The effective infection profile is shown where infectious students identified either by symptoms or by regular testing are isolated and thus, do not contribute to the infection profile. (*B*) Simulated temporal patterns of outbreaks with symptom screening and testing. Colors represent the mean class incidence rate (the number of new infections on a single day in each class divided by the class size) over the 500 simulations. (*C*) The final sizes of simulated influenza outbreaks under symptom screening and testing. Bars represent the upper 95% bounds, and the middle lines show the means over the simulations. Whiskers denote the upper 99% bounds. Note that symptom screening is also assumed to be conducted in the “regular tests” scenario. (*D*) The final size of simulated outbreaks with and without single-class closure strategies and the total days of class closures. Comparison of the cumulative number of infections with (*Upper*) and without (*Lower*) class closures in each setting. The distribution of the number of days of class closure is aggregated across the school. Bars represent the upper 95% bounds, and the middle lines show the means over the simulations. Whiskers denote the upper 99% bounds.

The infection profile constructed from the serial interval distribution used for the inference of the Matsumoto City data [mean: 2.2 d (52)] and the incubation period distribution of influenza A [median: 1.4 d (53)] suggested that screening by symptoms or regular tests may be less effective than for SARS-CoV-2 because the majority of infections may occur before isolation due to shorter infection cycles (Fig. 5*A*). In this setting, screening by symptoms and regular testing with 10 to 20% effective daily testing rates could reduce the reproduction number by only up to 30 to 40%—about half of what was estimated for SARS-CoV-2. Reflecting this, outbreak simulations with various interventions overall showed similar patterns to COVID-19, except that screening by symptoms/regular tests was estimated to be less effective for pandemic influenza than for COVID-19 (Fig. 5 *B* and *C*). Single-class closure strategies improved the outcome in most cases, with fewer days of class closure than in the COVID-19 simulation because a 5-d closure was sufficient to control an outbreak due to the short incubation period of influenza (Fig. 5*D*).

## Discussion

We examined the comparative effectiveness of school-based control measures for COVID-19 and pandemic influenza using an individual-level mathematical model that considers differential contact intensities within and between classes and year groups. We used a citywide primary school seasonal influenza epidemic dataset in Matsumoto City, Japan to parameterize the model and assessed a range of interventions, including screening, isolation, cohorting, and class closures. This dataset is one of the largest datasets of school outbreaks, containing over 10,000 students and 2,500 cases in a single epidemic season, which we believe provides the best available evidence on the detailed transmission patterns within schools.

Simulated school outbreaks of COVID-19 and pandemic influenza suggested two possible directions for management strategies. One of them involves a preemptive approach, which tries to reduce *R*_S_ before the emergence of an outbreak by interventions. If *R*_S_ is kept sufficiently small during everyday operation by incorporating various intervention methods (e.g., screening based on symptoms or regular tests and reducing outside-class interactions), a school would be resistant to sustained transmission. We propose that the preemptive approach should aim for *R*_S_ of at most around 0.5 such that the risk of large outbreaks (e.g., those involving over 10 secondary transmissions) is kept at a low level (∼10 to 20% or less), even with multiple introductions from outside school. This approach is likely to require combining multiple interventions if the baseline *R*_S_ is high; however, if successfully implemented, it may also ensure that schools can operate almost as normal, even amid ongoing community transmission. Alternatively, schools could also decide to operate with less stringent measures as a baseline and take a responsive approach as needed, where only students in classes with at least one confirmed case will isolate/quarantine (single-class closure). This strategy requires less intensive baseline measures and thus, could be more efficient and pragmatic in relatively low–community transmission settings. Moreover, it allows for ramping up control efforts according to the actual intensity of outbreaks (i.e., the scale of closure follows that of an outbreak). For the responsive approach to work, the outbreak ideally needs to be recognized before it spreads outside the initially affected class. Reduced outside-class interactions will assist this and are expected to reduce both the scale of outbreaks and class closures. While regular testing combined with the responsive approach could also bring a similar effect, it may not be recommended for COVID-19 because isolating only test-positive students (without class closure) could reduce the outbreak size without closing classes, which involves loss in education opportunity. That is, if a school can afford regular testing of students, the preemptive approach will allow sufficient control, and class closures may not be necessary. Alternatively, in such resource-abundant settings, intensive testing of a whole class where a positive case is found may achieve the same effect as a class closure in the responsive approach (54), which may be preferable in some settings as it allows uninfected students to remain at school.

When designing an overall school outbreak management plan, the epidemiological strengths and weaknesses of intervention measures should be evaluated. Regular testing is a powerful intervention that enables prompt detection and isolation of cases, which leave responsive class closures almost unnecessary. In our simulations, the effective daily testing rate of 10% exhibited sufficient performance in most cases. Using tests with a reasonably high sensitivity (∼70% or above), this means that the frequency of tests does not need to be more than once or twice a week. Although this would ease the required logistical burden, the option may not always be available to every school due to multiple aspects of resource constraints. The invasive nature of regular testing should also be recognized as students are a vulnerable population; less invasive methods, such as saliva tests, need to be considered (55). The issue of false positives also needs to be noted. An effective testing rate of 10% means that a 99.9% specific test may produce a false positive more than once a month on average, although this may be balanced by the reduction in the outbreak risk if only positive (including false-positive) students are isolated. Testing kits have different levels of specificity and should be selected considering the overall benefit given the risk of false positives. Meanwhile, screening by symptoms is less likely to suffer from this issue since it will be reasonable to ask students with obvious COVID-19–/flu-like symptoms to stay at home regardless of the actual cause. However, symptom lists for isolation should be carefully defined, particularly regarding mild symptoms [including those not typically considered as illness; e.g., loss of smell/taste for SARS-CoV-2 infection (48)], to balance between the risk of missing mild infections and the risk of isolating noninfectious students with mimicking symptoms (e.g., seasonal allergies). Given these benefits and limitations for different options, choice of intervention measures should be made considering epidemiological risk assessment and the current situation of community transmission as well as wider costs and practical constraints.

Several limitations must be noted for our analysis. First, there were potential sources of bias inherent to the nature of the Matsumoto City dataset, which we used for model calibration. Most importantly, the study was observational, and thus, the differences in the transmission patterns between schools of different class structures (class sizes and the number of classes per grade) might not be causal. We assumed that if class structures were altered by interventions, students would rewire their contacts according to the new class structure. However, students may respond differently in interventional settings in a pandemic. Empirical studies of reduced class sizes as an intervention against COVID-19 remain limited; those available reported little effect on COVID-19–related outcomes (56, 57), which is consistent with our findings, although these studies are also subject to limitations due to their observational nature. Moreover, the generalizability of the findings from primary schools in Japan, where students aged 6 to 12 spend most of the day with the same classmates, to other settings with different student ages or education systems needs to be carefully assessed. Reduced class sizes in pandemic settings may also be coupled with additional measures (e.g., physical distancing), which may provide mitigation effects not present in the Matsumoto City dataset.

Second, the epidemiological properties used in our simulation were subject to a number of assumptions. Within- and between-class/grade transmission patterns of COVID-19 and pandemic influenza were assumed to be proportional to those of seasonal influenza and scaled by the chosen *R*_S_ in the simulation. Shared main modes of transmission between COVID-19 and influenza do not necessarily imply that they exhibit identical dynamical behavior. However, modeling studies often use similar assumptions of proportionality between transmission and social contacts (34, 58), and we believe that our approach has strengths over such studies as it could indirectly measure social contacts in the context of transmission. Infections acquired from the household and the general community were not explicitly modeled and simply treated as external introductions. Overdispersion in the individual-level transmission was not considered in the main analysis because we hypothesized that the high degree of overdispersion observed for the general outbreaks of COVID-19 (31) may not necessarily apply to school settings with a smaller variation in contact behavior (59); we included an overdispersion parameter of 0.2 as a sensitivity analysis. Temporal profiles of infectiousness and the proportion of symptomatic infections were based on limited data (mostly from studies before the emergence of variants) and also, neglected individual-level variation. These may need to be updated in the future to reflect newer data; currently, the simulation results should be interpreted as a scenario analysis rather than conclusive predictions. The Omicron variant circulating as of early 2022 has been suggested to have a shorter generation time than previous variants (60–62). Although we did not include this variant in our simulation due to the limited epidemiological data, given the similarity of its generation time to that of influenza (2 to 3 d), our pandemic influenza simulation may be used as a useful reference.

Third, several potentially important elements in transmission dynamics have not been fully considered in our simulation. Students were assumed to only contact each other at school, and possible contacts outside of school on weekends or during closure/isolation were neglected as we assume that such contacts would not be encouraged during an outbreak. The number of nonhousehold contacts made by school-age children during the school closure in the United Kingdom in autumn 2020 (when the lockdown had been relaxed) has been reported to be disproportionally smaller than that after the school reopening (1 to 1.5 vs. 10 to 13 contacts per day) (63). The relative contribution of contacts between students outside school is thus likely limited in pandemic settings; schools could also encourage students to stay home during closures by, for example, offering online teaching. Transmission from teachers and staff was not considered. While the role of adults in overall dynamics has been more emphasized for COVID-19 than influenza (64), in recent studies it has been suggested that within-school transmission was more likely between students and between teachers (14, 65), and vaccination may also have reduced their relative role in school outbreaks of COVID-19. Preventing teachers or staff from being involved in superspreading events in school settings yet warrants further attention. Individual-level precautionary measures that are not expected to change the dynamical patterns of transmission (i.e., whose effect may be incorporated only by changing the assumed value of *R*_S_), including vaccines, universal masking, ventilation, etc., were not explicitly modeled and were assumed to be reflected by the value of *R*_S_ in the simulation. If these precautionary measures are in place, the results for a low *R*_S_ may apply even for a highly transmissible virus. Notably, COVID-19 vaccines have been given to children aged 5 to 11 y in a number of countries, including the United States as of November 2021, and other countries may also follow. If the vaccine uptake at school reaches a sufficient level, the target condition for the preemptive approach (i.e., *R*_S_ < 0.5) may be achieved without additional interventions. However, the global rollout of vaccine for children may take time due to a potentially different risk–benefit balance from adults; waning of immunity and the emergence of vaccine-resistant variants are also of continuing concern (66). We, therefore, believe that our assessment of a range of interventions and their combinations for school settings remains crucial.

The present study offers useful insights into the transmission patterns in school settings reflecting class/grade structures. We believe that these results not only inform modeling studies that incorporate transmission dynamics in schools but also aid in the planning and assessment of outbreak management strategies at schools for current and future pandemics.

## Materials and Methods

### Temporal Infection Profile of SARS-CoV-2 and Influenza.

We reconstructed the temporal infection profile of SARS-CoV-2 using distributions estimated by Ferretti et al. (44). Since the estimated infection profile used the date of symptom onset as a reference point and varied with the incubation period, we convolved the distributions of the onset-based infection profile and the corresponding incubation period to reconstruct the infection profile as a function of time from infection. The onset-based infection profile was estimated as a piecewise skew-logistic distribution in ref. 44; we convolved it with the incubation period distribution used in the same study, which was a mixture of lognormal distributions from the previous literature (67–73). Similarly, we obtained the infection profile for influenza by convolving the transmission hazard function we used in our Matsumoto City influenza study (43) [corresponding to a mean serial interval of 2.2 d (52)] and the estimated incubation period distribution of the H1N1 pandemic influenza (53).

The modification of infection profile *h_τ_* by screening was modeled as follows. Let *U_τ_* represent the survival function against screening (i.e., the probability that an infected individual remains undetected by day *τ* postinfection). The infection profile under symptom screening is represented as

[1] $$h\prime_{\tau }=h_{\tau }U_{\tau }=h_{\tau }{\left(1-\sigma \right)}^{\tau }\left(1-\varphi F_{\tau }\right),$$

where *σ* is the effective daily testing rate, *φ* is the proportion of transmission attributable to symptomatic infection, and *F_τ_* is the cumulative distribution of the incubation period. We assumed that students are regularly tested without a specific preference on the day of the week and that the number of students who undertake a test is thus uniformly distributed over a week.

### Simulation of COVID-19 and Pandemic Influenza Outbreaks in Schools.

We simulated school outbreaks using the estimated transmission patterns within and between classes/grades and infection profiles of SARS-CoV-2 and H1N1 pandemic influenza. Estimates of transmission risks within schools (i.e., the relative contribution of transmission within and between classes and grades) were retrieved from our previous study of seasonal influenza in Matsumoto City, Japan in the 2014/2015 season (43). The cumulative force of infection between a pair of students *i* and *j* over the course of infectiousness was modeled as a function of the class size *n* and the number of classes per grade *m*:

[2] $$\beta_{ij}=\beta_{d}n^{-\gamma_{d}}m^{-\delta_{d}},$$

where *d* represents the class/grade relationship between the students (i.e., whether they are 1] in the same class, 2] in the same grade but different classes, or 3] in different grades) and $\beta_{d}$, $\gamma_{d}$, and $\delta_{d}$ are the parameters estimated in ref. 43. The risk of a susceptible student *i* acquiring infection on day *t* is given as

[3] $$r_{t}^{i}=1-\exp\left(-\underset{j\in I_{t}}{\sum }\beta_{ij}h_{t-t_{j}}\right),$$

where *t_j_* is the date of infection for student *j* and *I_t_* represents a set of students infected by day *t*.

The within-school reproduction number *R*_S_ was defined as the sum of the pairwise cumulative force of infection from a student to all other students at school. For simplicity, we assumed that transmission risks between students are determined by class/grade structures and neglected the effect of other variables, such as sex, age, and individual-level precaution measures (therefore, grades in the simulations were only for labeling purpose and did not necessarily correspond to actual school years). The inference model used for the Matsumoto City data and posterior samples were used for simulation, where external infection from outside the school (i.e., transmission from households and the general community) was excluded except for the initial case.

For the baseline simulation, we assumed six year groups per school, with two classes (40 students each) per grade (i.e., *n* = 40, *m* = 2). Other combinations of *n* and *m* values were also used to assess interventions that change the class structures. Starting from a single initial case on a randomly chosen weekday, the simulation of transmission over 200 d was repeated 500 times, each with a different set of posterior samples of the parameters from ref. 43. Here, we limited our simulation to the within-school transmission dynamics, and students not attending school (e.g., on weekends or in isolation/quarantine) were assumed not to be involved in transmission.

We chose these values of *R*_S_ (2.0, 1.5, and 0.8) as representative of the Delta-like, Alpha-like, and the original SARS-CoV-2–/flu-like transmission potential, respectively. *R*_S_ was estimated to be around 0.8 for seasonal influenza (43), and we assumed the same value for the original SARS-CoV-2 strain because the basic reproduction number (*R*_0_) of COVID-19 is 1.5 to 2 times higher than seasonal influenza (64), while children were estimated to be about half as susceptible to infection (9). Studies suggested that the Alpha variant is ∼50% more transmissible (74) and also, has a relative risk of 1.2 for children compared with the original strain (30) and that Delta is ∼50% more transmissible than Alpha (75). However, these assumed values in our simulation should be viewed as hypothetical scenarios rather than accurate estimates. For each of the assumed values of *R*_S_, we rescaled the posterior samples of the relationship-specific transmissibility parameter *β_d_* such that the relative magnitude between *β_d_* is conserved and that their sum corresponds to the value of *R*_S_. Different types of interventions (Table 1) were incorporated into the simulation as follows. The *β_d_* values corresponding to different *n* and *m* were used to simulate the effect of changes in the size and the number of classes. Screening by symptoms and regular testing was implemented by using the modified infection profile in Eq. **1**. For reduced outside-class interactions (class cohorting) scenarios, we reduced *β_d_* values corresponding to outside-class interactions by either 50 or 90%. In addition to the “pure reduction” scenarios where outside-class interactions are reduced without countereffects, we also accounted for a possible compensatory increase in the within-class interactions. We assumed that within-class interactions may increase by 20% to compensate for a 50% reduction outside of class and by 40% to compensate for a 90% reduction.

Using the distribution of final outbreak size with a single initial case *q*_1_(*x*) obtained in the simulation, we also estimated the risk of large outbreaks (i.e., >10 and >30 secondary transmissions) given multiple introductions. The final outbreak size distribution given *z* introductions *q_z_*(*x*) is obtained as a *z*-fold convolution of *q*_1_(*x*):

[4] $$q_{z}\left(x\right)=\overset{x}{\underset{x_{1},x_{2},\ldots ,x_{z}=0}{\sum }}\delta_{\left(x\right)\left(\sum_{k=1}^{z}x_{k}\right)}\overset{z}{\underset{l=1}{\prod }}q_{1}\left(x-x_{l}\right),$$

where $\delta_{\left(x\right)\left(y\right)}$ is the Kronecker delta.

### Assessing the Risk of Undetected Spread of Infection.

We computed the distribution of the number of undetected infections by the detection of the first case in the school by sampling the date of detection in each of the 500 simulation results. Let *I_t_* be the number of new infections on day *t*. The cumulative distribution function (CDF) for the date of detection *T*_D_ is given as

[5] $$\text{CDF}\left(T_{\text{D}}\right)=1-\overset{T_{\text{D}}}{\underset{t=1}{\prod }}{\left({\left(1-\sigma \right)}^{T_{\text{D}}-t}\left(1-F_{T_{\text{D}}-t}\right)\right)}^{I_{t}}.$$

We sampled *T*_D_ according to this CDF and obtained the number of undetected infections as $\sum_{t=1}^{T_{\text{D}}}I_{t}-1$. The class that the first detected student belongs to was also sampled to provide the number of undetected infections outside that class, which was used to specify the spillover risk.

### Simulation of the Single-Class Closure Strategy.

The single-class closure strategy was simulated using the same approach as previously described, except that classes have either an “open” or “closed” state each day. Students in closed classes were considered to be isolating at home and thus, do not transmit to or receive infection from others on that day. For each infected student, the date of detection was sampled with the distribution in Eq. **5**, and the class closure started from the day after the first date of detection among the class. The class closure was assumed to last for 10 d (COVID-19) or 5 d (pandemic influenza). To assess the effectiveness of closure strategies, we compared the proportion of students experiencing infection by the end of the outbreak against the simulation results in the same settings but without closures.

All analysis was performed in Julia 1.5.2. Replication code is available in GitHub (https://github.com/akira-endo/schooldynamics_FluMatsumoto14-15).

## Supplementary Material

Supplementary File

## Data Availability

The replication code and posterior samples for the model simulation were retrieved from a previously published repository in GitHub (https://github.com/akira-endo/schooldynamics_FluMatsumoto14-15) (76).

## Footnotes

^*^The lower bound of 38% indicates the same transmissibility between symptomatic and asymptomatic infections, and 38% of total infections are assumed to be screened due to typical symptoms. The upper bound of 71% indicates that transmission from asymptomatic infection is 60% lower than that from symptomatic infection, and all symptomatic infections (50% of total infections) are assumed to be screened.
