# Supplementary material for: Simulating respiratory disease transmission within and between classrooms to assess pandemic management strategies at schools
Source: Proc Natl Acad Sci U S A. 2022 Sep 8;119(37):e2203019119. doi: 10.1073/pnas.2203019119 (PMC9478679; doi:10.1073/pnas.2203019119)
Supplement: Supplementary File [file pnas.2203019119.sapp.pdf]

PNAS
www.pnas.org

Supplementary Information for

Simulating respiratory disease transmission dynamics within and between classrooms to assess pandemic management strategies at schools

Akira Endo[1,2,3,4,5,*], CMMID COVID-19 Working Group[2], Mitsuo Uchida[6], Yang Liu[1,2], Katherine E. Atkins[1,2,7], Adam J. Kucharski[1,2], Sebastian Funk[1,2]

1. Department of Infectious Disease Epidemiology, London School of Hygiene & Tropical Medicine, London, WC1E 7HT, United Kingdom
2. The Centre for Mathematical Modelling of Infectious Diseases, London School of Hygiene & Tropical Medicine, WC1E 7HT, United Kingdom
3. The Alan Turing Institute, London, NW1 2DB, United Kingdom
4. School of Tropical Medicine and Global Health, Nagasaki University, Nagasaki, 852-8523, Japan
5. Japan Society for the Promotion of Science, Tokyo, 102-0083, Japan
6. Graduate School of Medicine, Gunma University, Gunma, 371-8511, Japan
7. Centre for Global Health, Usher Institute, University of Edinburgh, Edinburgh, EH16 4UX, United Kingdom

*Correspondence to: Akira Endo

This PDF file includes:

Supplementary text
Figures S1 to S8
SI References

Supplementary Information Text

Simulation results for pandemic influenza

Here we show additional simulation results for pandemic influenza (Figure S1). The main results were presented in the main text and the remaining results which showed almost equivalent patterns to the SARS-CoV-2 simulations are displayed for completeness. The distribution of undetected infections upon detection of the first case of pandemic influenza is shown in Figure S4A.

Peak weekly incidence under interventions

As an alternative outcome in school settings that focuses on operational burden, we compared the peak weekly incidence (i.e. the highest number of weekly cumulative cases over the course of an outbreak) of simulated COVID-19 and influenza school outbreaks (Figure S2), as opposed to final outbreak sizes compared in the main text (Figure 3). The results show qualitatively identical patterns to the final outbreak sizes, suggesting that interventions that reduce the final outbreak size could also reduce the peak incidence.

Temporal patterns of outbreaks under single-class closure strategies

Temporal patterns of incidence and class closure in simulated school outbreaks (Figure S3) suggest that, compared to the baseline (i.e. no intervention) scenarios in the main text (Figure 2A), an outbreak tends to be limited to a smaller number of classes under single class closure strategies.

Overdispersion and the risk of outbreaks

It has been suggested that the transmission of SARS-CoV-2 exhibit a high degree of individual-level variation (overdispersion) (1, 2). As a sensitivity analysis, we considered a negative-binomial overdispersion parameter $\kappa = 0.2$ in the simulation to account for potential superspreading. For each infectious student i, we rescaled the infectiousness variable w_i with a factor which follows a gamma distribution with a shape κ and a scale $1/\kappa$ (such that the mean is 1):

$$w_i' = \rho w_i, \qquad \text{(S1)}$$
$$\rho \sim \mathrm{Gamma}(\kappa, 1/\kappa).$$

In the presence of overdispersion, the risk of large outbreaks generally becomes smaller given the same reproduction number (Figure S4). However, the differences diminished with multiple introductions, and it was suggested that 10 introductions would pose an almost similar level of outbreak risks to the ‘no overdispersion’ scenario.

Class closure under intensive interventions

In the main analysis where we compared single-class closure strategy combined with class distancing, we excluded the potential compensatory increase in the within-class interaction. We reflected this effect on the class closure simulations where the inside-class interactions were increased by 20% and 40% respectively in scenarios with 50% and 90% reduction in outside-class interactions (Figure S5). With responsive class closures, increase in the within-class reproduction number hardly affected the outcome because the classes with at least one detected case was closed in the simulation regardless of how far infections had spread within the class, and further transmissions to outside the class were prevented.

Lower symptomatic proportion and closure strategies

We assumed a lower symptomatic proportion (25%) than used in the main analysis and assessed how it may affect the results of closure strategies. Both in the simulations of SARS-CoV-2 and pandemic influenza, lower symptomatic proportion resulted in larger outbreak sizes and broader class closure (Figure S6). Symptomatic screening becomes less likely to identify cases before they transmit and regular testing was suggested to be more effective.

Combination of class-changing interventions and responsive class closures

We assessed the effect of combining modified class structures (i.e. split classes and staggered attendance) and responsive class closures. Interventions changing class structures in addition to

the responsive class closure strategy did not exhibit clear incremental benefit (Figure S7). In the 'split classes' scenario, both the simulated outbreak size and the length of class closures increased.

Comparison of simulation outputs with observed school outbreak data in Texas, US.
To assess if our model outputs are in line with observed school outbreaks, we compared the outbreak sizes predicted in our simulation with COVID-19 outbreaks in elementary schools in Texas State, the United States. Texas Department of State Health Services publishes the reported number of weekly COVID-19 positive student cases from public schools in the state (3) (Figure S8A). We retrieved the cumulative number of COVID student cases between 2 August to 21 November 2021 from 3,495 elementary schools (enrolling a total of 1.9 million students) with at least one case during this period, when the Delta variant had been predominant (> 95%) in the United States (4). Mask mandates at schools were lifted in most school districts in Texas in summer 2021 following a governor's order prohibiting mask mandates at schools, although some districts defied this policy (5). As the dataset contained schools of various types other than elementary schools (e.g. middle and high schools), we selected schools that were identifiable as elementary schools (i.e. those whose abbreviated name in the dataset ends with "EL" or "ELEMENTARY"). The number of cases was suppressed in the dataset for privacy if the number was between 1 and 4; we randomly assigned a value 1–4 to schools with a suppressed cumulative case count. The mean number of students enrolled per school as of 29 September 2021 was 531 (interquartile range (IQR): 392–647), which was similar to the school size of 480 in our simulation. We neglected prior immunity against COVID-19 in students at those schools due to the small number of cumulative cases reported prior to 2 August 2021 (mean 2.1% of students enrolled; IQR 0.9–2.9). The attack rates of students in the schools included during the wave between 2 August to 21 November 2021 was of the same order of magnitude as the predicted outbreak size with the Delta-like school reproduction number of 2.0 under symptom screening (Figure S8B).

CMMID COVID-19 Working Group
Kaja Abbas, Kevin van Zandvoort, Nikos I Bosse, Naomi R Waterlow, Damien C Tully, Sophie R Meakin, Matthew Quaife, Timothy W Russell, Mark Jit, Anna M Foss, Alicia Rosello, Billy J Quilty, Kiesha Prem, Gwenan M Knight, Sam Abbott, Petra Klepac, Oliver Brady, Carl A B Pearson, Graham Medley, Samuel Clifford, Christopher I Jarvis, James D Munday, Frank G Sandmann, Fiona Yueqian Sun, Thibaut Jombart, Joel Hellewell, Hamish P Gibbs, Rosanna C Barnard, Rosalind M Eggo, Amy Gimma, Jack Williams, Nicholas G. Davies, Emily S Nightingale, Simon R Procter, W John Edmunds, Alicia Showering, Rachel Lowe, Katharine Sherratt, C Julian Villabona-Arenas, David Simons, Yung-Wai Desmond Chan, Stefan Flasche

CMMID COVID-19 Working Group funding statements
Kaja Abbas (B&MGF: OPP1157270), Kevin van Zandvoort (Elrha R2HC/UK DFID/Wellcome Trust/NIHR, DFID/Wellcome Trust: Epidemic Preparedness Coronavirus research programme 221303/Z/20/Z), Nikos I Bosse (Wellcome Trust: 210758/Z/18/Z), Naomi R Waterlow (MRC: MR/N013638/1), Sophie R Meakin (Wellcome Trust: 210758/Z/18/Z), Matthew Quaife (ERC Starting Grant: #757699, B&MGF: INV-001754), Timothy W Russell (Wellcome Trust: 206250/Z/17/Z), Mark Jit (B&MGF: INV-003174, NIHR: 16/137/109, NIHR: NIHR200929, European Commission: 101003688), Alicia Rosello (NIHR: PR-OD-1017-20002), Billy J Quilty (NIHR: 16/137/109, NIHR: 16/136/46), Kiesha Prem (B&MGF: INV-003174, European Commission: 101003688), Gwenan M Knight (UK MRC: MR/P014658/1), Sam Abbott (Wellcome Trust: 210758/Z/18/Z), Petra Klepac (Royal Society: RP\EA\180004, European Commission: 101003688), Oliver Brady (Wellcome Trust: 206471/Z/17/Z), Carl A B Pearson (B&MGF: NTD Modelling Consortium OPP1184344, DFID/Wellcome Trust: Epidemic Preparedness Coronavirus research programme 221303/Z/20/Z), Graham Medley (B&MGF: NTD Modelling Consortium OPP1184344), Samuel Clifford (Wellcome Trust: 208812/Z/17/Z, UK MRC: MC_PC_19065), Christopher I Jarvis (Global Challenges Research Fund: ES/P010873/1), James D Munday (Wellcome Trust: 210758/Z/18/Z), Frank G Sandmann (NIHR: NIHR200929), Fiona Yueqian Sun (NIHR: 16/137/109), Thibaut Jombart (Global Challenges Research Fund: ES/P010873/1, UK Public Health Rapid Support Team, NIHR: Health Protection Research Unit for Modelling Methodology HPRU-2012-10096, UK MRC: MC_PC_19065), Joel Hellewell (Wellcome Trust: 210758/Z/18/Z), Hamish P Gibbs (UK DHSC/UK Aid/NIHR: PR-OD-1017-20001, EDCTP2: RIA2020EF-2983-CSIGN), Rosanna C Barnard (European Commission: 101003688), Rosalind M Eggo (HDR UK: MR/S003975/1, UK MRC: MC_PC_19065, NIHR: NIHR200908), Amy Gimma (Global Challenges Research Fund: ES/P010873/1, UK MRC: MC_PC_19065), Jack Williams (NIHR Health Protection Research Unit and NIHR HTA), Nicholas G. Davies (NIHR: Health Protection Research Unit for Immunisation NIHR200929, UK MRC: MC_PC_19065), Emily S Nightingale (B&MGF: OPP1183986), Simon R Procter (B&MGF: OPP1180644), W John Edmunds (European Commission: 101003688, UK MRC: MC_PC_19065, NIHR: PR-OD-1017-20002), Alicia Showering, Rachel Lowe (Royal Society: Dorothy Hodgkin Fellowship), Katharine Sherratt (Wellcome Trust: 210758/Z/18/Z), C Julian Villabona-Arenas (BBSRC LIDP: BB/M009513/1), David Simons (BBSRC LIDP: BB/M009513/1), Stefan Flasche (Wellcome Trust: 208812/Z/17/Z)

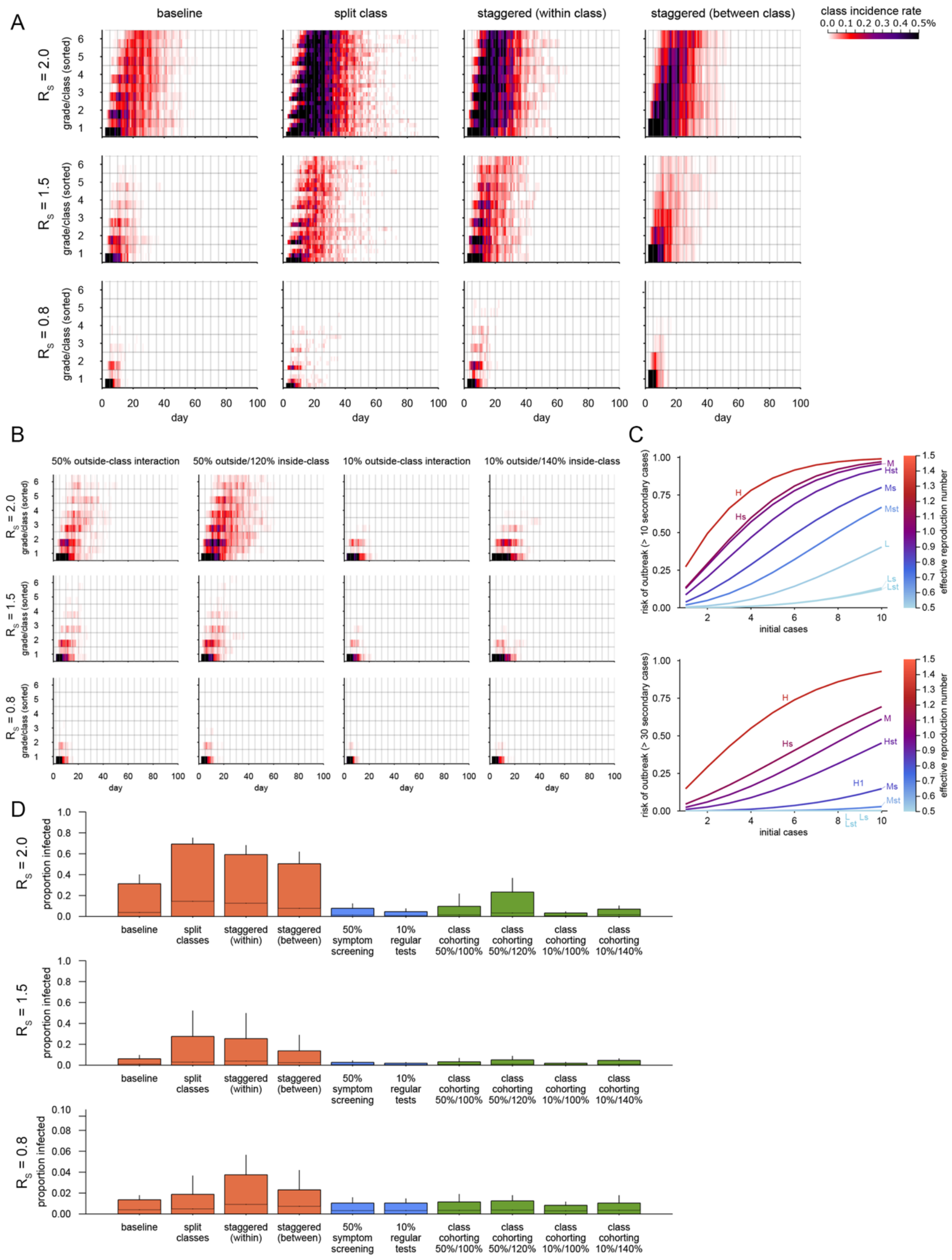

Fig. S1. Additional simulation results of pandemic influenza outbreaks. (A) Simulated patterns of outbreaks in schools under interventions changing class structures. Colours represent the mean class incidence rate (the number of new infections on a single day in each class divided by the class size) over the 500 simulations. (B) Simulations with reduced outside-class interactions. Compensatory increases in the within-class interactions (20% and 40% increase in within-class interactions to compensate 50% and 90% reduction in outside-class interactions, respectively)

were also considered as part of the simulation. (C) The estimated risk of large outbreaks with multiple introductions. Curves show the probability that the eventual number of secondary transmissions within school exceeds 10 or 30 cases in the intervention scenarios, given multiple introductions of infected student from outside the school. Interventions are labelled by the following notations. H: the school reproduction number (R_S) = 2.0; M: R_S = 1.5; L: R_S = 0.8; s: screening by symptoms; t: screening by regular testing (effective rate 10%). Colours denote the effective reproduction number within school for each intervention. (D) The distribution of simulated outbreak sizes under interventions. Bars represent the upper 95% bound and middle lines show the mean over the simulations. Whiskers denote the upper 99% bound. Colours represent different categories of interventions: changing class structures (orange), screening and testing (blue) and class cohorting (green). Note that symptom screening was also assumed to be conducted in the "10% regular tests" scenario.

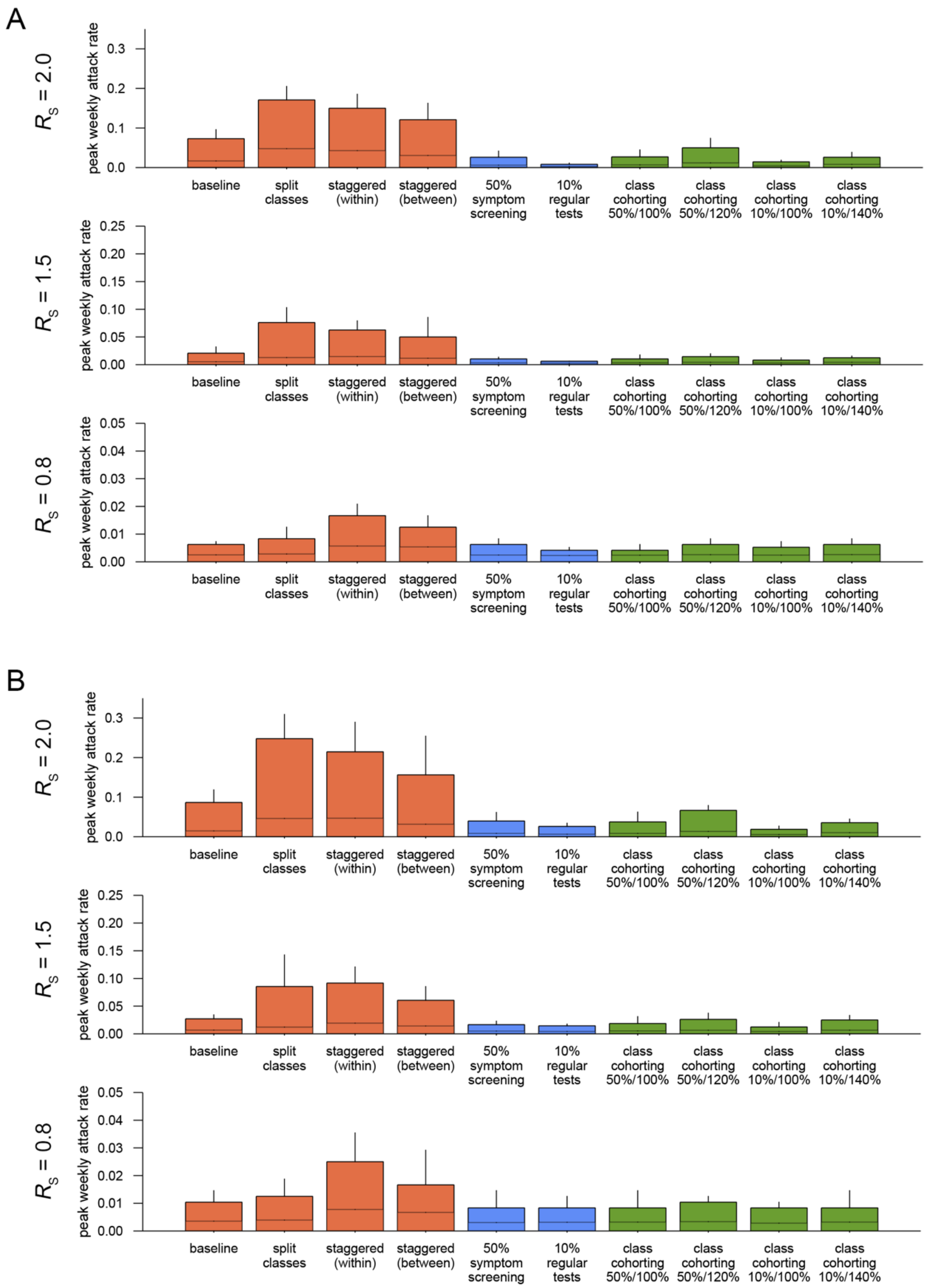

Fig. S2. The distribution of simulated peek weekly incidence of COVID-19 and influenza school outbreaks under interventions. Bars represent the upper 95% bound and middle lines show the mean over the simulations. Whiskers denote the upper 99% bound.

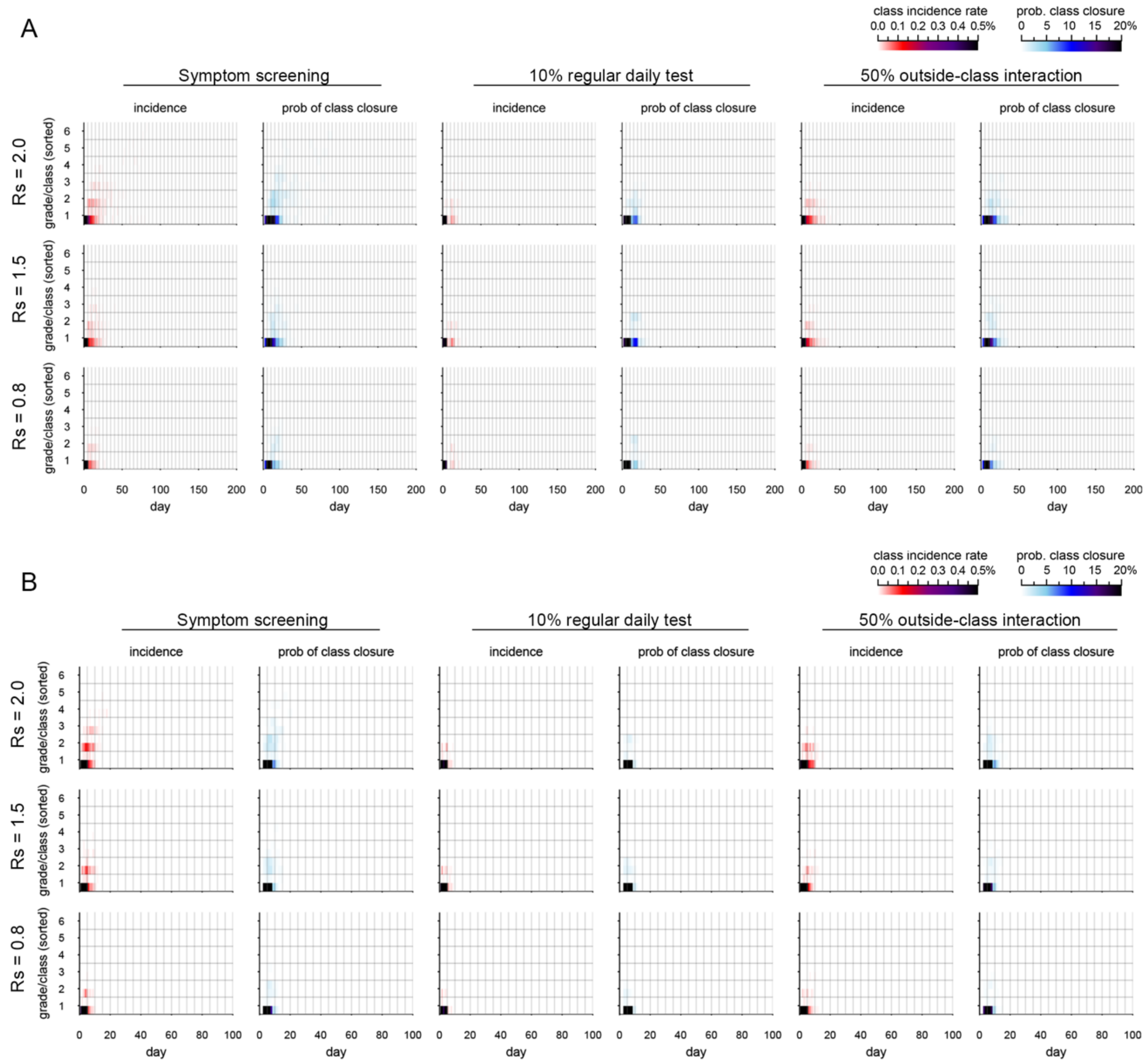

Fig. S3. Temporal patterns of simulated outbreaks under single-class closure strategies. Colours represent the mean class incidence rate (the number of new infections on a single day in each class divided by the class size) and the proportion of a class being closed over the 500 simulations. For each simulation, grades and classes are sorted by the date of the first case in the class so that the spread of infections in classes is time ordered from the bottom to the top. (A). Simulation of single-class closure strategies for SARS-CoV-2. (B). Simulation of single-class closure strategies for pandemic influenza.

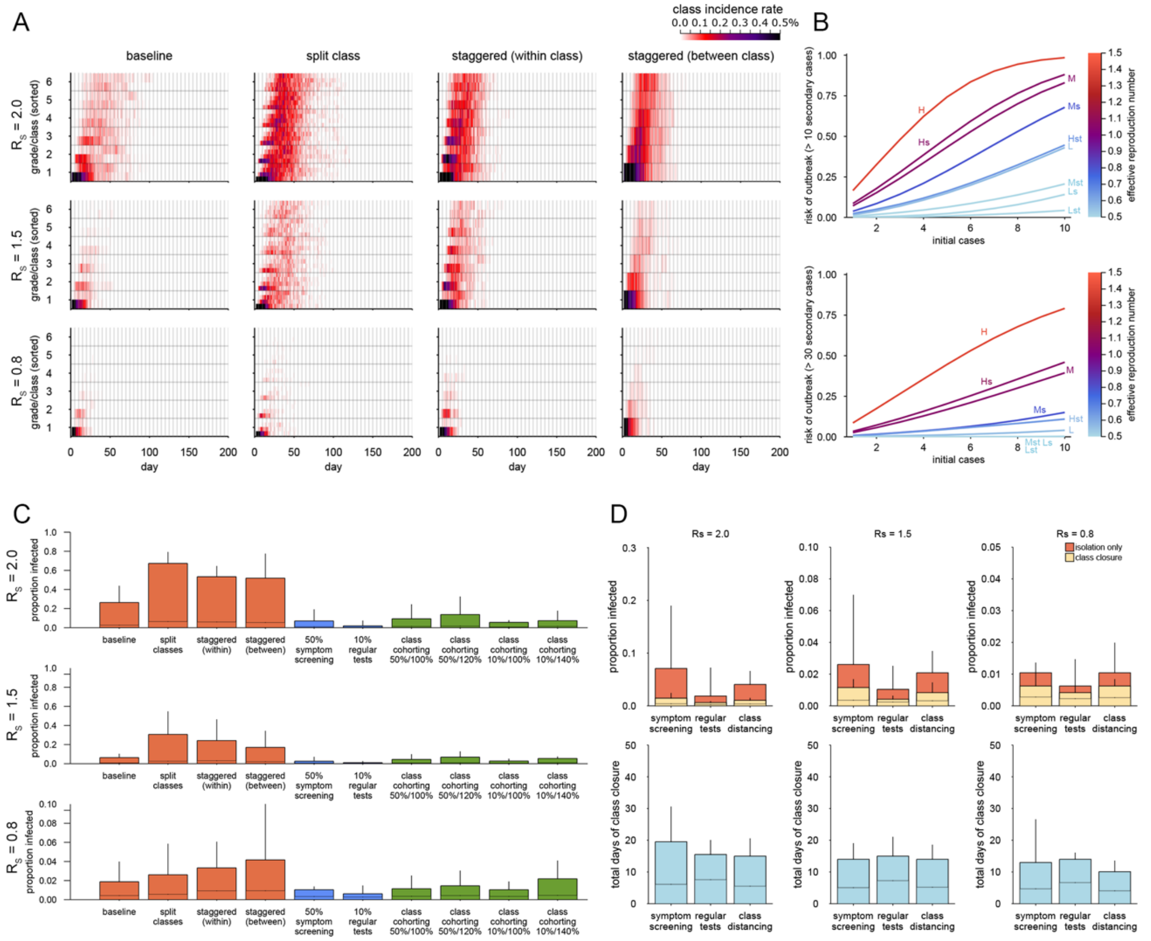

Fig. S4. Outbreak simulations accounting for overdispersion in SARS-CoV-2 transmission. (A) Simulated patterns of outbreaks in schools under interventions changing class structures. Colours represent the mean class incidence rate (the number of new infections on a single day in each class divided by the class size) over the 500 simulations. (B) The estimated risk of large outbreaks with multiple introductions. Curves show the probability that the eventual number of secondary transmissions within school exceeds 10 or 30 cases in the intervention scenarios, given multiple introductions of infected student from outside the school. Interventions are labelled by the following notations. H: the school reproduction number (R_S) = 2.0; M: R_S = 1.5; L: R_S = 0.8; s: screening by symptoms; t: screening by regular testing (effective rate 10%). Colours denote the effective reproduction number within school for each intervention. (C) The distribution of simulated outbreak sizes under interventions. Bars represent the upper 95% bound and middle lines show the mean over the simulations. Whiskers denote the upper 99% bound. (D) The final size of simulated outbreaks with and without single-class closure strategies and the total days of class closures. Top panels: comparison of the cumulative number of infections with and without class closures in each setting. Bottom panels: the distribution of the number of days of class closure aggregated across the school. Bars represent the upper 95% bound and middle lines show the mean over the simulations. Whiskers denote the upper 99% bound.

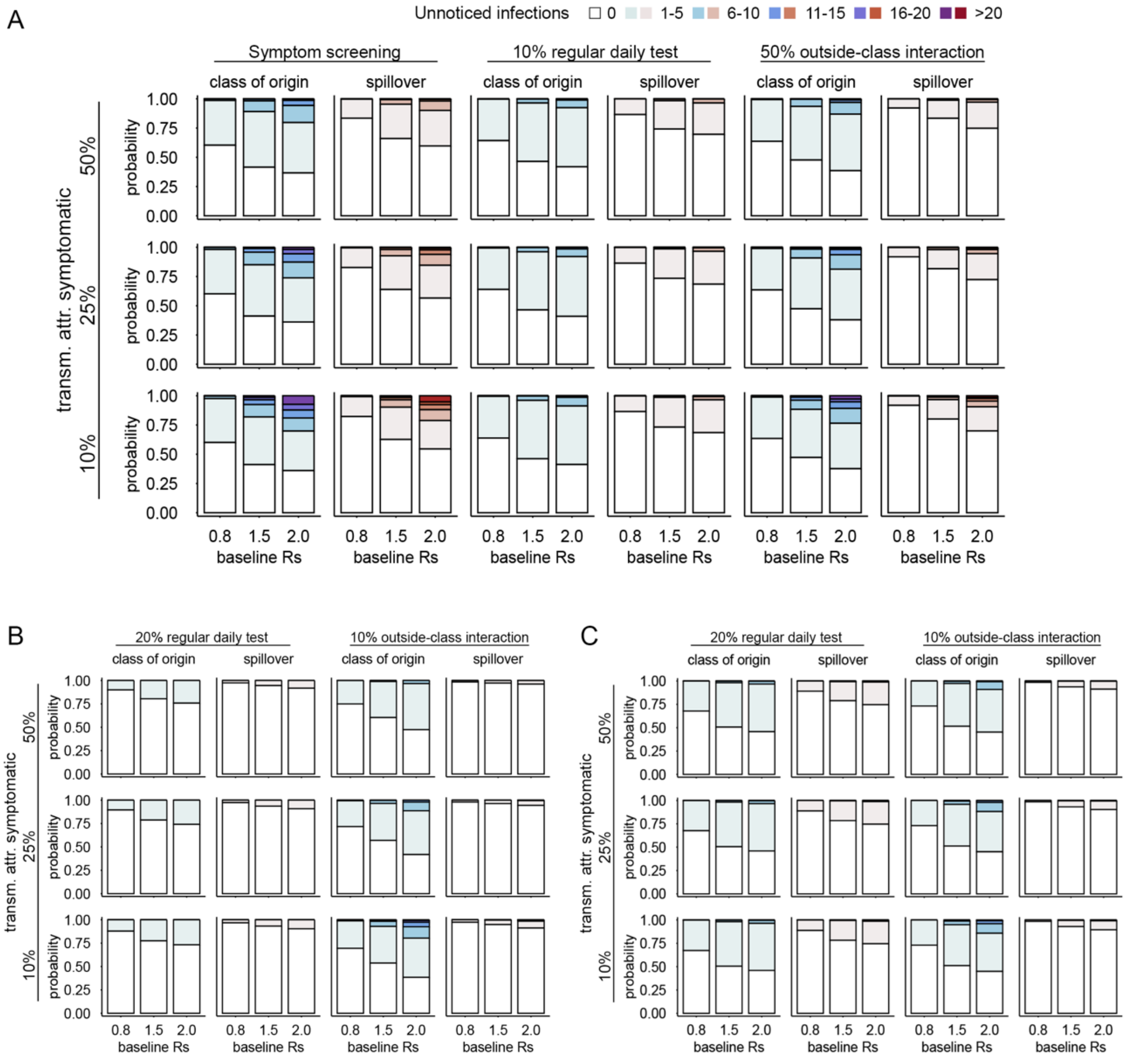

Fig. S5. Likely scales of pandemic influenza outbreak at recognition of a case and the effect of intensive interventions for COVID-19 and pandemic influenza. (A) The predicted distributions of the number of undetected infections with pandemic influenza by the first identification of a case (blue: overall; red: spillover infections outside the class of the initial case). (B) The predicted distributions of the number of unnoticed infections with SARS-CoV-2 by the first identification of a case with more intensive measures (regular testing with 20% effective daily rate/90% reduction in outside-class interaction). (C) The predicted distributions of the number of unnoticed infections with pandemic influenza by the first identification of a case with more intensive measures (regular testing with 20% effective daily rate/90% reduction in outside-class interaction).

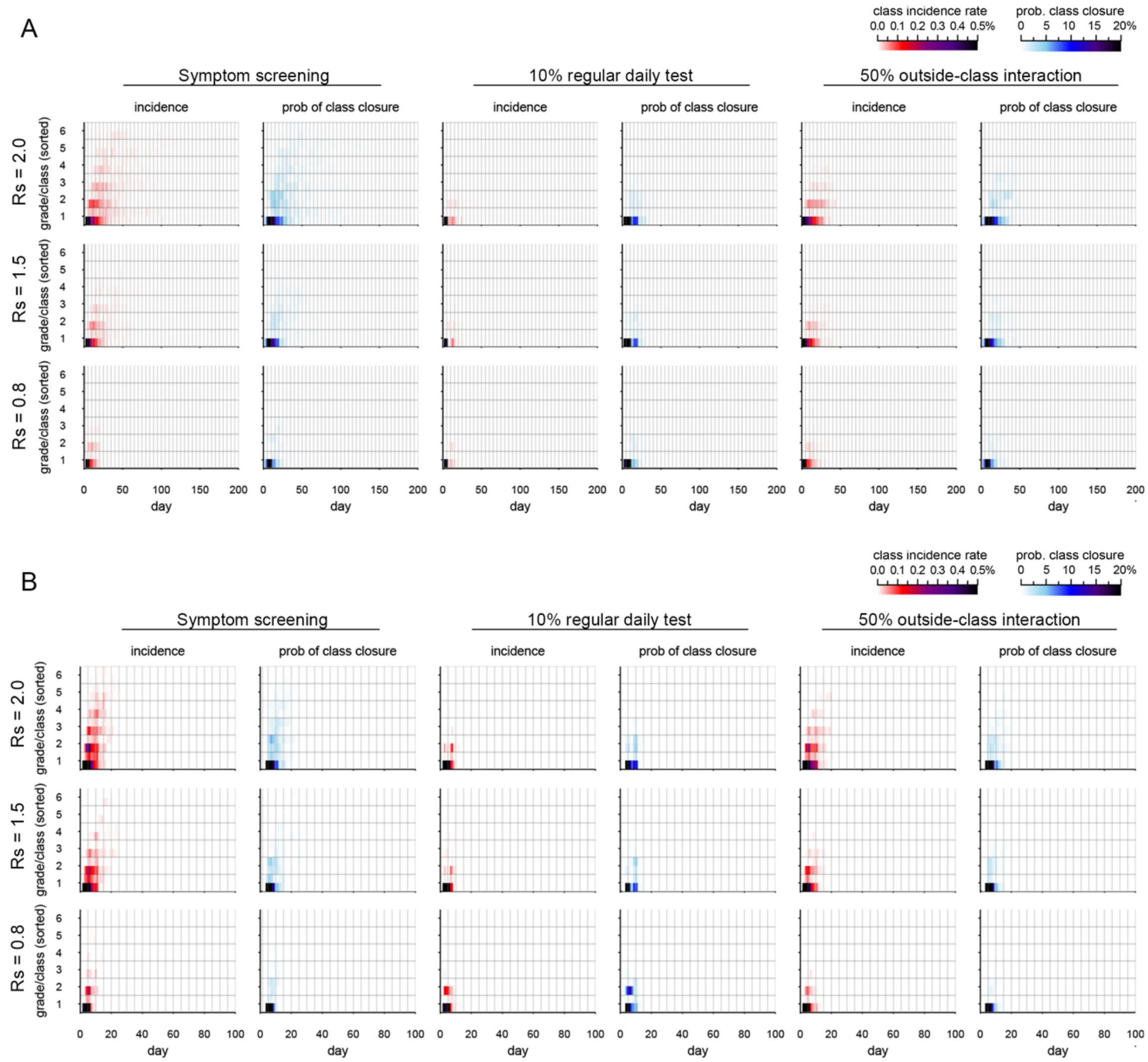

Fig. S6. Simulation of single-class closure strategies with a lower symptomatic proportion of 25%. Colours represent the mean class incidence rate and the proportion of a class being closed over the 500 simulations. (A). Simulation of single-class closure strategies for SARS-CoV-2. (B). Simulation of single-class closure strategies for pandemic influenza.

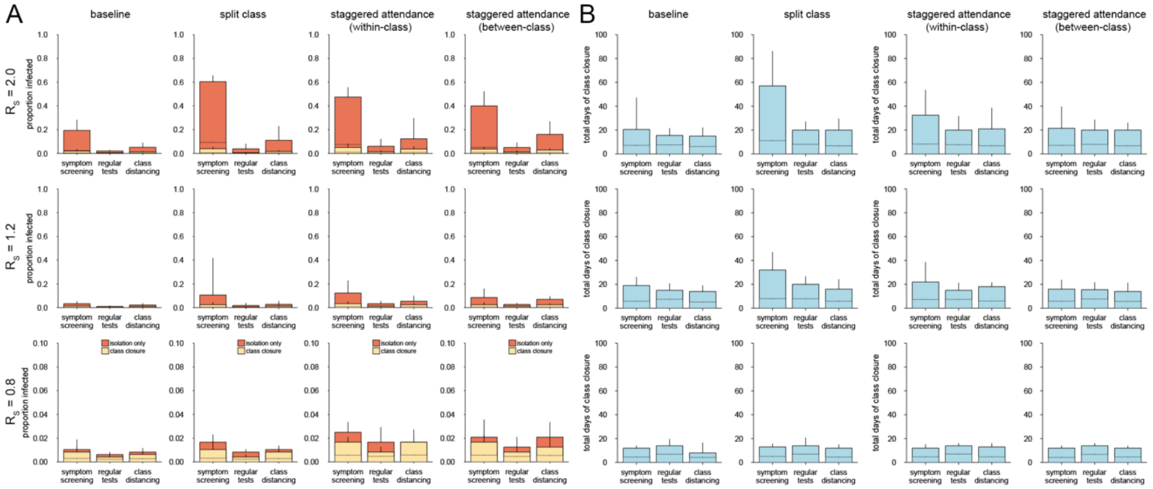

Fig. S7. Simulated outbreak sizes with and without single-class closure strategies combined with changes of class structures and total days of closures. (A) Comparison of the cumulative number of infections with and without class closures in each setting. Note that y-axes have different scales between the top and middle rows and the bottom row. (B) The distribution of the number of days of class closure aggregated across the school. Bars represent the upper 95% bound and middle lines show the mean over the simulations. Whiskers denote the upper 99% bound.

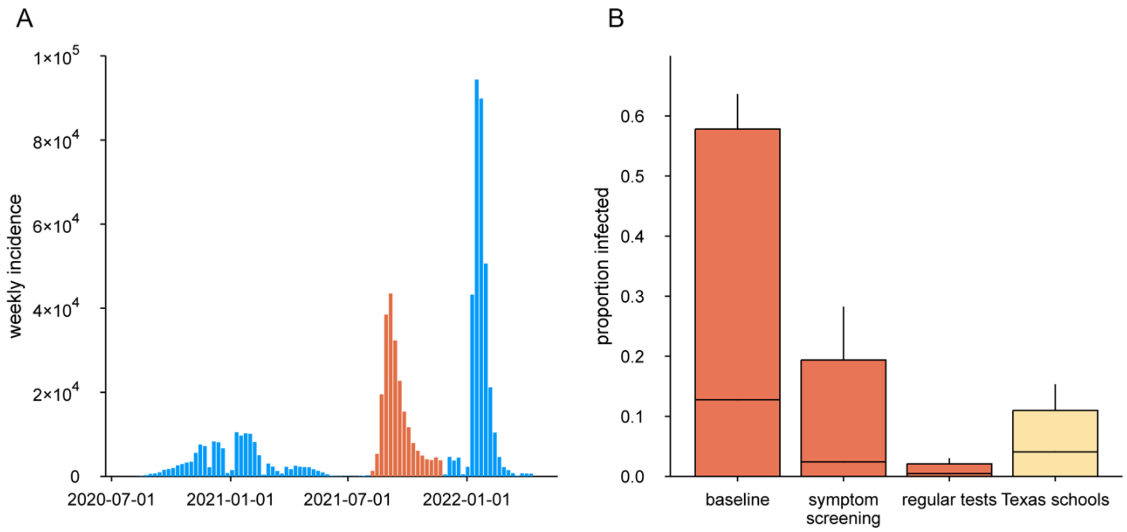

Fig. S8. School outbreaks of the Delta variant in Texas, US and simulated Delta-like outbreaks. (A) The observed weekly incidence of COVID-19 in elementary schools in Texas, US. The wave between 2 August to 21 November 2021 (coloured in orange), when the Delta variant had been dominant, was used for comparison with simulation. (B) Comparison between the sizes of simulated Delta-like school outbreaks and the observed Delta-variant outbreaks in Texas, US. Bars represent the upper 95% bound and middle lines show the mean over the simulations. Whiskers denote the upper 99% bound.

SI References

1. Q. J. Leclerc, N. M. Fuller, L. E. Knight, S. Funk, G. M. Knight, What settings have been linked to SARS-CoV-2 transmission clusters? *Wellcome Open Research* **5**, 83 (2020).

2. A. Endo, S. Abbott, A. J. Kucharski, S. Funk, Estimating the overdispersion in COVID-19 transmission using outbreak sizes outside China. *Wellcome Open Research* **5**, 67 (2020).

3. Texas Department of State Health Services, Texas Public Schools COVID-19 Data (Accessed 17 April, 2022). https://www.dshs.texas.gov/coronavirus/schools/texas-education-agency/

4. A. S. Lambrou, Genomic Surveillance for SARS-CoV-2 Variants: Predominance of the Delta (B.1.617.2) and Omicron (B.1.1.529) Variants — United States, June 2021–January 2022. *MMWR Morb Mortal Wkly Rep* **71** (2022).

5. D. Huey, Texas school COVID-19 cases: How those with mask mandates compare to those without. *Kxan Austin* (2021) (Accessed 17 April, 2022). https://www.kxan.com/investigations/
